# Severe Insect Pest Impacts on New Zealand Pasture: The Plight of an Ecological Outlier

**DOI:** 10.1093/jisesa/ieaa018

**Published:** 2020-04-23

**Authors:** Stephen L Goldson, Gary M Barker, Hazel M Chapman, Alison J Popay, Alan V Stewart, John R Caradus, Barbara I P Barratt

**Affiliations:** 1 AgResearch, Christchurch, New Zealand; 2 Bio-Protection Research Centre, Lincoln University, Lincoln, New Zealand; 3 Landcare Research, Lincoln, New Zealand; 4 School of Biological Sciences, University of Canterbury, Christchurch, New Zealand; 5 AgResearch, Palmerston North, New Zealand; 6 PPG Wrightson Seeds, Lincoln Christchurch, New Zealand; 7 Grasslanz Technology Ltd., Palmerston North, New Zealand; 8 AgResearch, Invermay Agricultural Centre, PB, Mosgiel, New Zealand

**Keywords:** exotic weevil pests, New Zealand pasture, biotic resistance importation biocontrol, conservation biocontrol

## Abstract

New Zealand’s intensive pastures, comprised almost entirely introduced *Lolium* L. and *Trifolium* L. species, are arguably the most productive grazing-lands in the world. However, these areas are vulnerable to destructive invasive pest species. Of these, three of the most damaging pests are weevils (Coleoptera: Curculionidae) that have relatively recently been controlled by three different introduced parasitoids, all belonging to the genus *Microctonus* Wesmael (Hymenoptera: Braconidae). Arguably that these introduced parasitoids have been highly effective is probably because they, like many of the exotic pest species, have benefited from enemy release. Parasitism has been so intense that, very unusually, one of the weevils has now evolved resistance to its parthenogenetic parasitoid. This review argues that New Zealand’s high exotic pasture pest burden is attributable to a lack of pasture plant and natural enemy diversity that presents little biotic resistance to invasive species. There is a native natural enemy fauna in New Zealand that has evolved over millions of years of geographical isolation. However, these species remain in their indigenous ecosystems and, therefore, play a minimal role in creating biotic resistance in the country’s exotic ecosystems. For clear ecological reasons relating to the nature of New Zealand pastures, importation biological control can work extremely well. Conversely, conservation biological control is less likely to be effective than elsewhere.

The primary industries of New Zealand have been identified as having a ‘fundamentally important role’ in the country’s economy (New Zealand Treasury Office 2016). The most significant of these is pastoral agriculture (dairy, beef, sheep, and deer sectors) which in 2011 had an estimated gross agricultural production value of NZD19.6 billion (New Zealand Treasury Office 2016). Fourteen million hectares, of the nation’s total land area of 26.8 million hectares, are given over to grassland farming (Beef and Lamb New Zealand 2018). Significantly, c.7 million hectares of this area are intensively farmed and comprise pastures made up largely of a mixture of two exotic grassland species, perennial ryegrass (*Lolium perenne* L.) (Poales: Poaceae) and white clover (*Trifolium repens* L.) (Fabaceae: Fabales), thereby creating highly modified landscapes. Indeed, these species are so important to the New Zealand economy that ryegrass is listed as the most valuable plant species in New Zealand, with clover as the third most important (Craig 2016).

This contribution discusses the origin and extent of invasive pest impacts on New Zealand pasture and contrasts these with those found in the northern native grasslands of the ‘Holarctic.’ Here the term ‘Holarctic’ is used generically to designate the combination of the vast Northern Hemisphere areas comprising the Nearctic (North America), Western Palaearctic and the Eastern Palaearctic regions (e.g., Greathead and Greathead 1992) combined. The Nearctic and Palaearctic land-masses have been linked intermittently by the Bering land-bridge and consequently, the faunas are relatively closely related.

While New Zealand’s pastoral landscapes may look superficially similar to many pasturelands elsewhere (as the result of attempts by colonizers to replicate European farming systems) their origins and ecological contexts are extremely different. It is the differences that have very major implications for the severity of pest impacts.

Extensive pest damage led to decades of intensive New Zealand research into pasture pest management that comprised three distinct phases. The first phase, before World War 2, was primarily focused on two indigenous species, the New Zealand grass grub (*Costelytra giveni* Coca-Abia and Romero-Samper) (formerly C. *zealandica* (White)) (Coleoptera: Scarabaeidae) and the porina moth complex (comprising seven *Wiseana* spp.) (Lepidoptera: Hepialidae). Although indigenous, these pests can be effectively regarded as invaders of a new grassland habitat consisting of Holarctic plant species (e.g., Cockayne 1911). At the time suggested methods of control included mass-trapping of flying adults, various forms of cultivation, tilth management, changes to sward composition and cultivation rotations including cropping (Cockayne 1911, 1920). The available insecticides included lead arsenate, carbon disulphide, naphthalene, calcium cyanide, and white arsenic. While these were of little practical value, at the time excellent progress was made in understanding the pests’ ecologies as exemplified by Dumbleton (1942).

The second phase of New Zealand’s pasture pest management began with the use of DDT and other organochlorine insecticides in 1947 (e.g., Boul et al. 1994). This was discussed by Kelsey and Hoy (1950), who began DDT trials in that year against both *C. giveni* and *Wiseana* spp. and recommended that it be applied in combination with superphosphate. During the 1950s and 1960s organochlorine compounds revolutionized the control of practically all of the soil-dwelling pasture pests and, given their efficacy, ecological research into pasture pests waned (e.g., Given 1968, Pottinger 1973).

The third phase started in the early 1970s when DDT and other organochlorine insecticides were banned (Boul et al. 1994). Thereafter, no completely satisfactory alternative chemical control was found. The organophosphates tested were short-acting and often acutely toxic (Trought 1980). These circumstances led to a very significant and sustained phase of research based on the creation of a government-mandated ‘insect pest task force’ (Pottinger 1975). This resulted in a significant recruitment of entomologists and their ensuing intensive research reverted back to New Zealand pasture pest biology, building on the earlier work of Dumbleton (1942) and others. There was a particular emphasis on pest population dynamics, modeling, and key-factor analysis. Since then, plant resistance, the development of biologically based pesticides and advances in parasitoid-based biological control have also become prevalent (e.g., Ferguson et al. 2018).

However, despite 100 yr of investigation into how to suppress broadacre (i.e., typically land parcels greater than 4,000 m^2^) invasive insect pests in New Zealand, they continue to cause recurring and severe pasture pest problems (e.g., Goldson et al. 2016).

## Origin and Nature of New Zealand Pastures

All ecosystems have changed substantially since the last Ice Age, particularly from human impacts. These modifications have included significantly altering plant and animal distributions, the arrival of invasive species and species extinctions (e.g., Vitousek et al. 1997). However, in the Holarctic, much of its fundamental and diverse ecology (Dengler et al. 2014) has persisted; this is quite unlike New Zealand, where very recent and vast ecological changes have occurred. New Zealand’s indigenous grassland and forest ecosystems not only differ ecologically from their Holarctic equivalents, they are globally unique, reflecting geographic isolation and complex geological and human settlement history. The archipelago began its split from Gondwanaland 85 million years ago and has been geologically isolated for at least 55 million years. During this time, dramatic geomorphic and climatic transformations (Bunce et al. 2009) had profound effects on New Zealand’s native fauna and flora, shaping its forest and grassland ecosystems (e.g., Perry et al. 2012, Wilmshurst et al. 2014). These ancient evolved ecosystems have been supplemented with more recent species immigration (e.g., Gaskin 2006). Prior to Polynesian settlement c.700 yr ago, the islands were mostly covered in a dense, evergreen forest. Grassland and shrubland landscapes were largely confined to the small, semiarid interior of the southern South Island, unstable riverbeds, cliffs, sand dunes, and the extensive upper montane regions of the Southern Alps (McGlone 1989) and montane regions of the North Island, often disturbed by volcanism. The mostly endemic tall tussock grass genus *Chionochloa* Zotov, dominated many of these grasslands (Cockayne 2011) with other genera including *Poa* L. and *Festuca* L., as well as herbs and shrubs such as *Aciphylla* J R Forster & G. Forster spp. and *Dracophyllum* Labill spp. Subsequently, Polynesian fires (Wilmshurst et al. 2011) may have reduced forest cover by as much as 40%, the drier eastern districts becoming largely denuded of forest (Perry et al. 2012). In place of the forest, extensive fernlands and grasslands proliferated (McGlone 2001, McGlone et al. 2005). In the montane areas the most successful native grassland colonizers of burnt-off forest habitat included *Chionochloa* spp. with short tussock grasses, (e.g., *Festuca novae-zelandiae* (Hack.) Cockayne (Lord 1993, Cockayne 2011) and *Poa cita* Edgar (synonym *P. lanceolata* (G. Forst.) Hook. Ex Speg.). In its unaltered state lowland grassland would have had a significantly higher proportion of short tussocks (Connor and Vucetich 1964) and both *F. novae-zelandiae* and *P. cita* remain major components of lowland degraded short tussock grassland today (Rose and Frampton 2007). However, New Zealand’s pre-European settlement native short tussock grasslands would have looked significantly different to today’s short tussock grassland (Lord 1993), as they would have had a rich indigenous herb, fern and shrub layer (Godley 1967). The unique lack of grazing mammals from ancient New Zealand has been a key factor in the evolution of such ecosystems.

The arrival of European settlers in the early 1800s saw the introduction of various ruminants (Molloy 1977), and by the 1850s, pastoral farming was well-established in New Zealand (O’Connor 1986). Further, in response to a local and global demand for timber, the settlers felled much of the remaining lowland forests, thereby creating further pastoral and cropping farms. By 1874 sheep numbers reached 10.7 million and rabbit populations had reached plague proportions (O’Connor 1986). Within 150 yr, after the arrival of European settlers in the middle of the 19th Century, the 60–65% of the original forest that was left undisturbed by Māori was progressively reduced to the current cover of c. 23%.

New Zealand’s now vitally important, intensively farmed pastures are based on a partial transplant of a very small number of Holarctic species. The plant species sown in New Zealand represent <1% of the numerous leguminous and graminaceous species found across the Holarctic. By far, the largest contribution to New Zealand forage production is made by perennial ryegrass (*L. perenne* L.) and annual ryegrass (*L. multiflorum* Lam.) cultivars and hybrids (Craig 2016). However, in addition, there is relatively minor contribution by other plant species including some prairie grasses (*Bromus* Scop. spp.), cocksfoot (*Dactylis glomerats* L.), fescues (tall fescue, *Schedonorus arundinaceus* (Schreb.) Dumort., and meadow fescue, *Schedonorus pratensis* (Huds.) P. Beauv.) (Clough and Hay 1993). The predominant legumes include cultivars of white clover (*T. repens* L.) and red clover (*T. pratense* L.) (Clough and Hay 1993).

In recent years, smaller proportions of pasture herbs such as chicory (*Chicorum intybus* L.) and plantain (*Plantago lanceolata* L.) have contributed to a slightly more diverse set of pastures. Weedy Gramineae in New Zealand’s intensive pastures may include bent grasses (*Agrostis* L. spp.) and in the north particularly, *Poa annua* L. along with some C4 subtropical grasses such as kikuyu (*Pennisetum clandestinum* Hochst. Ex Chiov.) and paspalum (*Paspalum dilatatum* Poir. and *P. distichum* L.) (Lambrechtsen 1972).

The high productivity of New Zealand’s pastures (Moot et al. 2009) has been realized through the use of fertilizers, irrigation, plant and animal genetics and management that includes optimal paddock subdivision and grazing regimes. Further, new technologies are adopted by astute and educated farmers/land managers who rapidly take up the results of scientific research (Moot et al. 2009, Caradus et al. 2013). For example, as part of creating and maintaining elite pastures, close attention has been paid to breeding *Lolium* spp. cultivars in combination with selected optimal strains of pest-protective *Epichloë festucae* var. *lolii* Latch, M.J. Chr. and Samuels (Hypocreales: Clavicipitaceae) endophytes as discussed below by Johnson et al. (2013). This and other plant breeding technology, have resulted in the high use of propriety cultivars of introduced species of grass and clover sown in New Zealand pasture because of clear commercial advantages (Caradus et al. 2013).

The scientific literature specifically pertaining to New Zealand’s pasture ecosystem function and biodiversity per se is relatively scant. Laliberté et al. (2013) measured trait convergence and divergence in grasslands along gradients of primary productivity and disturbance at both local and metacommunity scales. Concern has been increasing about the persistence of sown species in New Zealand pastures (e.g., Daly et al. 1999, Tozer et al. 2014) and its association with pest impacts, climate change, intensified grazing, and other factors including the spread of invasive weed species (Bourdôt et al. 2007). When pastures become open, weedy, and no longer meet feed requirements of livestock, they are resown with high-performing cultivars. Significantly, 5–7% (0.5 million hectares) of intensively cultivated pasture area is renewed annually (Moot et al. 2009), sometimes in a rotation that includes annual forage crops such as maize or brassicas.

Two features make New Zealand pasture uniquely vulnerable to pest damage: 1) pastures’ distinct lack of evolved complexity resulting in a narrow range of unrelated plant species and 2) the phylogenetic remoteness of the introduced forage plants from New Zealand’s native flora and fauna. The latter is well exemplified by the *Lolium* and *Festuca* genera. Both belong in the tribe Poaceae, subtribe Loliinae and are evolutionarily very close (Cheng et al. 2016). The Loliinae subtribe has two main divisions, broad-leafed (e.g., broad-leafed *Festuca* spp. and all *Lolium* spp.) and narrow-leafed (e.g., inclusive of *Festuca* spp.) (Edgar et al. 2000, Darbyshire and Warwick 2008, Inda et al. 2008, Minaya et al. 2017). Molecular evidence based on nuclear and plastid markers show that broad-leafed and narrow-leafed lineages diverged approximately 20 mya in Eurasia (Minaya et al. 2017). Current hypotheses propose that the narrow-leafed Loliinae arrived in Australasia via the Americas 11–5.3 mya and then, in another long-distance dispersal, both broad- and narrow-leafed members of the Loliinae subtribe arrived in Australasia from Eurasia approximately 5.3–2.6 mya (Minaya et al. 2014, 2017). Following these introductions, narrow-leafed *Festuca* species have speciated in New Zealand, resulting in about 13 indigenous narrow-leafed *Festuca* species. However, no broad-leafed *Festuca* spp. native to New Zealand exist at all (Edgar et al. 2000, Inda et al. 2008, Minaya et al. 2017). Thus, New Zealand native narrow-leafed *Festuca* species are phylogenetically very remote (20 my) from the broad-leafed *Lolium* species that were naturalized in New Zealand in the mid-19th Century. Very significantly clovers were also absent in New Zealand’s indigenous ecosystems (e.g., Allan et al. 1961).

## Species Diversity and Mobility Across Holarctic Grassland/Wooded Boundaries, Compared to the New Zealand Boundaries and the Implications for Biological Control

### Holarctic

As part of a fully evolved complex ecosystem, the diversity and distribution of grassland insect species across ecosystems are far greater in the Holarctic than in New Zealand. For example, in the Holarctic, Dengler et al. (2014) found up to 98 species of vascular plants in 10 m^-2^ of Transylvanian meadow. Also, in these Holarctic ecosystems, the same species of plants and animals are often found distributed across adjacent forest and grassland (e.g., Ellis et al. 2014) reflecting their widespread mobility (e.g., Dengler et al. 2014, Ellis et al. 2014, Kirby and Watkins 2015). Although farming is extensive over wide areas of the Holarctic, such land-use occurs in vast gaps in what is still primitively a sylvan landscape (Kirby and Watkins 2015, Goldson et al. 2017).

### New Zealand

In contrast to the Holarctic, there is minimal movement of New Zealand’s native plants and animals in and out of exotic pasture; rather, they remain in the country’s indigenous forests and grasses, or close to pasture boundaries (e.g., Wratten and Pearson 1982, Topping and Sunderland 1992, Sivasubramaniam et al. 1997, Topping and Lövei 1997, Harris and Burns 2000, Barlow and Goldson 2002, Mclachlan and Wratten 2003, MacLeod and Moller 2006, Moller et al. 2008, Goldson et al. 2014a, Tomasetto et al. 2017, Curtis et al. 2019). For example, in his survey of the beetles in the natural and modified ecosystems in Lynfield, Auckland, Kuschel (1990) found only 9.1% of the beetle species recovered from ‘open fields’ (paddocks, urban parks, recreational fields, and densely planted gardens) were endemic. The converse applied with respect to exotic invertebrates in the indigenous ecosystems. Here, Kuschel (1990) noted that 21.6% of the beetles in the bush were exotic. Similarly, in a montane area of New Zealand, Ewers and Didham (2008), collected beetles across the edge gradients of fragmented remnants of native beech forest (*Fuscospora* spp. (R. S.Hill and J. Read) Heenan and Smissen and *Lophozonia* spp. Turczaninow) set within a matrix of extensively grazed grasslands. In total, 26,312 individuals were collected comprising 769 beetle species (98% native to New Zealand) from across seven edge gradients. This research showed that the native species declined sharply in the grassland areas despite containing at least some native grasses and herbs that would have been evolutionarily less remote to the native insect species than the ryegrass/clover found in intensively farmed pasture. Consistent with this lack of movement of native insect species, observations by Brockerhoff et al. (2010) indicated that New Zealand’s indigenous forest ecosystems have been subjected to very little invasion by exotic species. The obvious exception being the devastating incursion of forests by the wasps *Vespula germanica* (F.) and *V. vulgaris* (L.) (Hymenoptera: Vespidae) (e.g., Harris 1991, Harris et al. 1991, Beggs and Rees 1999) which have been driven by opportunistic exploitation of honeydew.

Topping and Lövei (1997) compared spider diversity in New Zealand native tussock grassland versus that in immediately adjacent exotic sown pasture (typically being ryegrass/clover). Remarkably, they found that all of the species collected from the tussock grassland site were native, except for an exotic unidentified theridiid (Araneae: Theridiidae) that also occurred in the pastoral habitat. Thus, although the native grasslands are possibly structurally close to some agricultural habitats, differences in phylogenetic histories of New Zealand’s exotic and native grassland ecosystems discussed above, effectively exclude spider species overlap.

Such generalized lack of movement of indigenous natural enemy species beyond their native ecosystem boundaries in New Zealand is unsurprising given the tens of millions of years of separation between the country’s indigenous ecosystems and anything resembling grazed pasture now. Significantly, as a result of this relative immobility, the majority of the indigenous natural enemies contribute little, if any, suppression of invasive species within New Zealand’s pastures.

## Uniquely Severe Pest Impacts in New Zealand’s Intensive Pasture

In view of the nature of New Zealand’s pasture ecosystems, there are extensive and severe pests impacts. Ferguson et al. (2018) estimated that in an average year, the economic impact on farmers alone, is between NZD 1.7B and NZD 2.3B.

Clearly, such impacts are related to the exotic pasture’s low plant and animal diversity coupled with their phylogenetic remoteness from New Zealand’s native species, including a complete absence of indigenous clover (Allan et al. 1961). The resulting minimal biotic resistance has led to ongoing pest invasions such that there are now 2,200 exotic invertebrates in New Zealand (Barlow and Goldson 2002). Furthermore, the repeated history of pest outbreaks strongly points to enemy release (e.g., Liu and Stiling 2006, Mills 2017, Schulz et al. 2019) in spite of controversy about the generalized application of the theory (e.g., Colautti et al. 2004). In New Zealand’s case the idea of enemy release is supported by many of the invasive species in New Zealand pasture causing far more damage than in complex ecosystems elsewhere (e.g., Goldson et al. 2005a, 2016; Heimpel and Mills 2017; Ferguson et al. 2018). Linked to such exotic pest outbreaks in New Zealand’s pasture is the persistence of relict behaviors. Examples of this include photoperiodically induced diapause in *L. bonariensis* (Kuschel) (Coleoptera: Curculionidae) (e.g., Goldson and Emberson 1980, Goldson et al. 1984), the flight patterns of this species (Barker et al. 1989b, Goldson et al. 1999). The same applies to the aestivatory and flight behavior of the lucerne pest, *Sitona discoideus* Gyllenhal (Coleoptera: Curculionidae) (Goldson et al. 1988). Such behavioral phenomena, apparently offer little, if any, selective advantage in New Zealand’s pasture and climate, pointing to a history of minimal selection pressure. Thus, the usual assumptions about adaptive fitness and species competition do not necessarily hold in the New Zealand pasture ecosystem.

By way of illustration of the susceptibility of New Zealand pasture to exotic pest species invasion, there are three well-worked examples of weevils that are of relatively minor significance in their native ecosystems but cause severe damage to New Zealand’s exotic grassland forages. The first is a destructive pest of Gramineae, *L. bonariensis,* which was first recorded in New Zealand in 1927 (Marshall 1937), the second is the clover root weevil, *S. obsoletus* (Gmelin), (Coleoptera: Curculionidae) identified in New Zealand in 1995 by Barratt et al. (1996) and the third is a lucerne pest, *S. discoideus*, that was first described in New Zealand by Esson (1975).

### Listronotus bonariensis

Exactly when *L. bonariensis* established in New Zealand is uncertain, but it is likely that it, being a grassland species, was present in the grasses or hay used as stock feed on steamships (the first in 1846) between South America and New Zealand prior to the 20th Century (Williams et al. 1994), and/or in the early trade in pasture seeds (Brooking and Pawson 2007, Brooking and Pawson 2010). In New Zealand’s North Island pasture, the weevil has been reported to reach densities of up to 723 adults per m^2^ (Barker and Addison 1993). The species’ economic impact on New Zealand agriculture can be up to NZD200 million p.a. (Prestidge et al. 1991, Ferguson et al. 2018). This damage is well-explained by the *L. bonariensis* larval stages killing 3–8 tillers per developed adult (Barker et al. 1989a). Such *L. bonariensis* densities and plant damage levels are far higher than those that occur in the weevil’s primitive habitat in the ‘vega’ or ‘mallines’ ecosystems in Argentina (Lloyd 1966). Typical native grass species of the mallines, are *Festuca pallescens* (St.Yves Parodi, *Poa lanuginosa* Poir. and *Hordeum comosom* J. Presl and C. Presl (Gaitán et al. 2011). These Gramineae are genetically close to some of New Zealand’s introduced Gramineae including common cereals and pasture grasses (Morrison 1938, Jacques 1940, Doull 1954, Kain and Barker 1966). It is, therefore, unsurprising that L. *bonariensis* is associated with these economically important plants. Barratt et al. (2016) have also made the point that *L. bonariensis* has been able to adapt and often thrive on many other host grasses (e.g., Barker 1989).

In its native range, *L. bonariensis*, presumably, with its co-evolved cohort of South American natural enemies, occasionally inflicts damage to grasses and cereals, particularly *L. multiflorum* (A. J. Popay, personal communication), but with no indication of complete pasture grass loss such as observed in New Zealand (e.g., Parra et al. 2017). Also, the severe impacts of *L. bonariensis* in New Zealand pasture are in stark contrast to what has been found in the complex, evolved grassland ecosystems in Europe. In spite of five interceptions of *L. bonariensis* in the European Union, which has suitable conditions for its establishment, there are no reports of its presence (Jeger et al. 2018).

### Sitona obsoletus

The second example of catastrophic pest invasion into New Zealand pasture has been *S. obsoletus*. Although first identified in 1995 (Barker and Addison 1996, Barratt et al. 1996), it had been present in the New Zealand Waikato region since 1994 (Barker and Addison 1996). This species feeds only on *Trifolium* spp. (Murray and Clements 1994) and had immediate and serious impacts on New Zealand clover. In the northern North Island, shortly after establishment, populations of root-feeding *S. obsoletus* larvae ranged from 1291 m^-2^ to 1400 m^-2^ (Willoughby and Addison 1997, Gerard et al. 2010). Again this contrasts starkly to densities found in its native range (e.g., Mowat and Clawson (1996) found peak larval densities of c.30 m^-2^).

Larval survival of *S. obsoletus* increases linearly with increasing availability of rhizobial root nodules, which provide food and protection for the vulnerable first instar larvae (Gerard 2001, Gerard et al. 2010). Apart from and a congeneric invader, *S. discoideus* in New Zealand lucerne, *Medicago sativa* L. (see below), no other invasive insect species have been found that preferentially uses the root nodule niche thereby allowing the invading *Sitona* spp. to establish with minimal competition. Again, the damage potential of *S. obsoletus* in New Zealand is very high (Ferguson et al. 2018) with entire clover populations eliminated from areas of New Zealand pasture and consequent impacts on forage nutritive quality and persistence (Eerens et al. 2002, Gerard et al. 2007). The environmental implications of this have been intensified through increased compensatory use of nitrogenous fertilizers (Ferguson et al. 2018). Ferguson has estimated the cost to New Zealand farmers to be up to NZD225 million p.a.

### Sitona discoideus

The third example, *S. discoideus* that was first observed in Hawkes Bay in 1974 (Esson 1975) and it thereafter dispersed rapidly throughout New Zealand wherever lucerne occurred. The phenology and population dynamics of this weevil have been described by Goldson et al. (1984 and 1988) who showed that the species exhibited univoltine aestivatory behavior. At the same time it rapidly became apparent that the species was damaging (Goldson et al. 1984) with mid-season dry-matter yield reductions of >46% (Goldson and Muscroft-Taylor 1988) mainly through larval mining of nitrogen-fixing rhizobial root nodules and destruction of root hairs (Goldson et al. 1984) in a way very similar to that of *S. obsoletus* in *T. repens* (Gerard et al. 2010). Peak larval densities of *S. obsoletus* depended on soil moisture levels with numbers exceeding 5,000m^-2^ on occasions (Goldson et al. 1986). With damage thresholds occurring as low as 1,200 to 2,100 larvae m^-2^ (depending on soil moisture conditions) yield losses frequently occurred (Goldson et al. 1985). Adult defoliation was also often very apparent, but it was difficult to separate these effects from the impacts of larval feeding (Goldson et al. 1984). Further, Goldson and Muscroft-Taylor (1988) noted that these effects were particularly prevalent on light free-draining soils. Where soils are deep and more retentive of mineralized nitrogen, *S. discoideus* are found to be less abundant and damaging. This is taken to be the result of the plants’ reduced immediate dependence on atmospherically-fixed nitrogen and consequent lower levels of nodulation which in turn supported fewer larvae (Goldson and Muscroft-Taylor 1988, Barlow and Goldson 1990). There have been no estimates of the national cost of impact of this species but it is likely to be in the tens of millions (NZD) (Goldson and Muscroft-Taylor 1988).

These three cryptic and highly invasive weevil species found in New Zealand pasture and forage highlight the perennial need for excellent border biosecurity, particularly as they are so difficult to detect (Goldson et al. 2016). This urgency is brought into sharp relief when it is recognized that there are c.100 other *Sitona* spp. in Europe (de Castro et al. 2007) and 117 *Listronotus* spp. in temperate South America (Donato et al. 2003). In addition to the examples of invasive species above and as discussed earlier, small number of native insect species (viz. *C. giveni* and *Wiseana* spp.) have adapted to New Zealand’s nutrient-rich exotic pastures causing severe and persistent damage (Ferguson et al. 2018). Further, even though closely related to *C. giveni*, *C. brunneum* (Broun) has not made the transition into the exotic pastures (Lefort et al. 2013). Lefort et al. (2013) found that *C. giveni* has a preexisting ability to tolerate plant defence chemicals that *C. brunneum* does not have and this explains why *C. giveni* has become a serious pest of pasture throughout New Zealand (Lefort et al. 2015a,b). Further relating to the *Wiseana* spp. and C. *giveni* damage, Parker et al. (2006) have also shown, through meta-analysis of numerous manipulated field studies, that exotic plants can be sometimes be particularly badly damaged by native herbivores, because the introduced plants have no evolved defences. This would certainly seem to apply to at least some of New Zealand’s exotic pasture species.

## Biological Control in Holarctic Grasslands and New Zealand Pasture

The nomenclature and terminology for biological control has recently been thoroughly discussed in the literature by Heimpel and Mills (2017). In general, ‘biological control’ is an ecosystem service in which a pest is effectively controlled through interactions with natural enemies. Relevant to this contribution and as discussed below, the two types of biological control referred to are ‘importation biological control’ (often also called ‘classical biological control’) and ‘conservation biological control.’

## Importation Biological Control

### Holarctic

The subject of Holarctic importation biological control is covered in the comprehensive review of Heimpel and Mills (2017) and the contributions of several other authors (e.g., Greathead 1986, Greathead and Greathead 1992, Clarke and Walter 1995, Cock et al. 2016). Notably, Greathead and Greathead (1992) and updated by Cock et al. (2016), developed the BIOCAT database, which contains comprehensive world-wide records of introductions of natural enemies for the control of pests. This BIOCAT database makes useful reference to biogeography. In general though, the importation biological control biological literature rarely makes specific reference to pests of grasslands in the Holarctic. As an approximation, however, if consideration is extended to cereals, grains and forage legumes, then there is reference to importation biological control in the Holarctic. Heimpel and Mills (2017) made useful note of such examples although significantly, this is often about sourcing Holarctic natural enemies for release in the non-Holarctic zones rather than the reverse. An example of forage pest biocontrol was the introduction of *M. aethiopoides* from North Africa to suppress *Sitona cylindricollis* Fahraeus (Coleoptera: Curculiondae) in Canada (Loan and Holdaway 1961) and *Hypera postica* (Gyllenhal) (Coleoptera: Curculionidae) in the United States (Coles and Puttler 1963). Subsequently, it was introduced into New Zealand in 1982 (Stufkens et al. 1987) where it has reduced *S. discoideus* in New Zealand to below damage threshold levels (e.g., Barlow and Goldson 1993). Evans et al. (2006) reported on the successful suppression of the cereal leaf beetle, *Oulema melanopus* (L.) (Coleoptera: Chrysomelidae), in western North America using *Tetrastichus julis* (Walker) (Hymenoptera: Eulophidae) indigenous to Europe. This species has a very high reproductive output and can respond rapidly to target pest buildups and, therefore, provide useful control in ephemeral habitats such as cereals (Evans et al. 2006).

In addition to these contributions, in North America there is an array of curculionid species very similar to *L. bonariensis* that attack amenity grasses. These include the annual blue grass weevil, *Listronotus maculicollis* Kirby (Coleoptera: Curculionidae), the bluegrass billbug, *Sphenophorous parvulus* Gyllenhal (Coleoptera: Curculionidae), and the hunting billbug, *S. venatus vestitus* Chittenden (Coleoptera: Curculionidae). The biology, ecology, and management of these species have been well summarized by Vittum et al. (1999).

There has also been considerable attention to the biological control of the pea leaf weevil, *Sitona lineatus* (L.) (Coleoptera: Curculionidae) in Britain and North America which has features in common with *S. discoideus*. For example, Vankoski et al. (2011) discussed the potential and actual contribution of indigenous natural enemies of *S. lineatus* in western Canada and drew attention to the cosmopolitan species, *Bembidion quadrimaculatum* (L.) (Coleoptera: Carabidae) as a potentially useful predator. However, there was no specific reference to any importation biological control. With regard to pulse crops, Knodel and Shrestha (2018) have comprehensively reviewed impacts of wireworms (Coleoptera: Elateridae) and cutworms (Lepidoptera: Noctuidae) and noted significant effort to develop the use of biopesticides (in particular nematodes) but again, no reference was made to importation biological control.

Thus overall, there is a paucity of importation biological control literature associated with Holarctic grassland ecosystems and this may be interpreted to reflect a lack of need for such intervention.

### New Zealand

The often root-feeding, stem-mining, and fossorial habits of New Zealand’s pasture pests along with their widespread distribution has meant that the use of synthetic insecticides remains neither economically nor environmentally feasible (Barlow and Goldson 2002, Ferguson et al. 2018). An exception to this is the limited use of such pesticides to protect of seedlings in newly sown pasture seedlings (Barker et al. 1991, Addison et al. 1993, Ruppert et al. 2017).

Under such circumstances the most practical broad-acre pasture pest management options have come down to biological control and *Epichloë* endophyte-based plant resistance (see below) (e.g., Goldson et al. 2005a).

In particular, importation biological control has been used extensively against the three major weevil pests in New Zealand’s pastures as described earlier. In all cases, this was based on host native-range searches for control agents, in temperate South America (*L. bonariensis*), Europe (*S. obsoletus*), and North Africa (*S. discoideus*). This led to the identification of three koinobiontic wasps in the genus *Microctonus* Wesmael (Hymenoptera: Braconidae). Those selected were *M. hyperodae* Loan against *L. bonariensis* (Goldson et al. 1990a), *M. aethiopoides* Loan (Irish strain) against *S. obsoletus* (Gerard et al. 2011) and *M. aethiopoides* Loan (Moroccan strain) against *S. discoideus* (Aeschlimann 1983, Stufkens et al. 1987). Of these *M. hyperodae* and *M. aethiopoides* (Irish strain) were parthenogenetic, whereas the Moroccan strain of M. aethiopoides, active against the lucerne weevil, reproduces sexually. Generally, parasitism by these species was found to occur at low levels in their native ranges. Goldson et al. (1990b) found that the prevailing level of parasitism of *L. bonariensis* by *M. hyperodae* in temperate South America was c.5%, although often the sampled weevil populations showed no parasitism at all (S. L. Goldson, unpublished data). However, there was one occasion in January 1989 when 77% parasitism was measured in a weevil population collected in La Serena Chile, although the sample size was not recorded. Loan and Lloyd (1974) observed *L. bonariensis* parasitism of 39% in Bariloche, Argentina in October 1972. McNeill et al. (2006) reported parasitism levels of less than 8% in *S. obsoletus* by *M. aethiopoides* (Irish strain) in western Ireland and Aeschlimann (1978) never reported parasitism levels greater than 31% in *M. aethiopoides* (Moroccan strain) in the Mediterranean area.

These generally low observed parasitism rates in the native ranges, which may at least in part be related to host scarceness, contrast markedly with what has been found in New Zealand where parasitism rates by all three parasitoids had reached >90% within 1–3 yr after release (Barlow and Goldson 1990, Barker and Addison 2006, Gerard et al. 2011). In all cases such levels demonstrably reduced or eliminated pest damage (e.g., Barlow and Goldson 1993, Barker and Addison 2006, Goldson et al. 2011, Barker 2013, Basse et al. 2015, Ferguson et al. 2018).

The consistency of such a triple success is extraordinary. Based on historical analysis by Gurr and Wratten (1999), imported biological programs have an estimated chance of 10% chance of success. That all three pasture biological control initiatives were successful, meant that the odds of achieving such a result was 1:1,000. Therefore, a recurring question is why has this been the case? Again, the answer points strongly to the unique and very invasion-prone ecology of New Zealand’s exotic grassland ecosystems. It is highly likely that the control agents benefitted from the same enemy release as the target pest species. For example, in spite of very close attention during the mass-rearing of least 1.3 million *M. hyperodae* there was never any evidence at all of any hyperparasitism (e.g., McNeill et al. 1999, 2002).

With regard to the New Zealand populations of exotic pests and introduced exotic biological control agents, it is reasonable to expect that they would typically exhibit bottlenecked genetic diversity, genetic drift, and possible reduced adaptive capability. However, such assumptions may presume that the date of first recorded observation (or the deduced time of first incursion) was the only establishment event. However, it is likely that a species like *L. bonariensis*, that probably first arrived a century ago (Marshall 1937), have undergone unrecorded serial reintroductions and now comprise greater diversity than that found in more recently arrived pest populations. Further, such arrivals could well have come from a number of geographical areas, not only from across their native range. Williams et al. (1994) used Random Amplified Polymorphic DNA (RAPD) marker analyses to conclude low genetic diversity in *L. bonariensis* but recent ongoing work using next-generation sequencing (GBS) has indicated that this is probably not the case (Jacobs 2019).

## New Zealand Importation Biological Control Collapse

It is against this background of success that the biological control of *L. bonariensis* by *M. hyperodae* then failed after c.14 generations (Goldson and Tomasetto 2016; Tomasetto et al. 2017, 2018a). There were indications this had been the result of adaptation by the weevil resulting from selection pressure by the parasitoid (Goldson et al. 2014b, Goldson and Tomasetto 2016, Tomasetto et al. 2017). This interpretation built on an earlier suggestion by Goldson et al. (2015a) that the decline was the result of selected-for enhanced weevil evasive behavior. This idea was subsequently supported by Tomasetto et al. (2018b). These authors observed that between 1993 and 2018 there had been a significant decline in the slope of the type 1 parasitism functional response curve based on a manipulated range of weevil densities. This significant decline reflected increased evasive behavior by the weevil in 2018 compared to that in 1993 and overall, indicated that behavioral change can rapidly generate new phenotypes (Sih et al. 2011). With regard to the effect of global warming day-degree data collected between these dates do not point to significant changes in the parasitoid / host phenology (M. W. Shields, personal communication).

Despite the biological control failure in the *M. hyperodae*/*L. bonariensis* system, in general, the susceptibility of pests to importation biological control is known to be very stable. This is conferred by several factors as follows. Host susceptibility to biological control agents can be stabilized through the provision of either spatial (Hanski 1981) or temporal refugia from the control agents (Godfray et al. 1994). Similarly, low disturbance regimes are also known to preserve biological control effectiveness (Jonsson et al. 2012), as do diverse agroecosystems that often comprise a wide range of natural-enemy guilds (Tylianakis and Romo 2010). Additionally, Turnock and Muldrew (1971) suggested that the likelihood of resistance is reduced in the presence of more than one effective control agent and experimental results have supported this (Kraaijeveld et al. 2012). Significantly, the unique nature of New Zealand pastoral ecosystems is such that none of these factors are found in the *L. bonariensis*/*M. hyperodae* dynamic. Added to this and as discussed earlier, the nature of New Zealand’s pastoral ecosystem has resulted in very high weevil and parasitoid populations leading to uninterrupted host-specific selection pressure. Finally, *M. hyperodae* parthenogenetic reproduction had meant that the sexually reproducing *L. bonariensis* has had the ability to adapt to avoid parasitism, whereas the parthenogenetic parasitoid has largely been unable to counteract. This dynamic is sometimes referred to as ‘an unequal evolutionary arms-race’ (Kraaijeveld 1994, Henter 1995, Henter and Via 1995). Consistent with this, Casanovas et al. (2018) empirically modeled the *L. bonariensis*/*M. hyperodae* interaction based on field- and laboratory-derived parameters obtained from earlier long-term studies. This work strongly supported the idea that resistance is inevitable when hosts have more genetic variance (due to either fewer bottlenecks or greater sexual recombination rates) than their predator. Their model found that unless the parasitoids have at least three times the genetic diversity of the host, then some level of host resistance would develop. Conversely, in the case of a sexually reproducing parasitoid attacking a sexually reproducing weevil, the same model predicted no host resistance (Casanovas et al. 2018). This is what has been found in the case of *M. aethiopoides* (Moroccan) parasitizing *S. discoideus* in lucerne over the last 35 yr (S. L. Goldson, unpublished data). The uniqueness of these results in New Zealand pasture has been highlighted and discussed by Pennisi (2017) and Mills (2017).

The only other published example of acquired host resistance to a parasitoid is that of an exotic field cricket (*Teleogryllus oceanicus* (Le Guillou)) (Orthoptera: Gryllidae) in Hawaii, which through selection pressure, stopped stridulating (after about 24 generations) because such activity attracted the parasitoid fly *Ormia ochracea* (Bigot) (Diptera: Tachinidae) (Pascoal et al. 2014). Further research showed that this cessation of stridulation had occurred via different genetic changes in populations collected from two separate islands within the archipelago.

## Conservation Biological Control

### Holarctic

Given the entirely contrasting histories of the Holarctic grasslands and those of New Zealand’s intensive pastures, there are commensurately stark differences in the potential for conservation biological control between these two areas. This is unsurprising given that it is the plant biodiversity in pasture habitats and surrounding environments that ultimately contribute to ecosystem services and function (e.g., Altieri and Nicholls 2018, Shields et al. 2018). In the Holarctic there are numerous natural enemy species that can provide pest suppression and may be manipulated or enhanced for such purpose. Here the grazed and cultivated areas are surrounded by species-diverse areas such as woodlands, field margins, permanent grasslands and hedgerows. They represent a fully evolved and complex macro-arthropod predatory fauna, some of which are listed in Table 1.

**Table 1.** Summary of diverse macro-arthropod predatory fauna in the Holarctic (e.g., Edgar and Loenen (1974), Dennis and Fry (1992), Downie et al. (1999), Cole et al. (2005), Woodcock and Pywell (2010))

Order	Family
Coleoptera	Carabidae, Staphylinidae, Coccinelidae, Cantharidae, Drillidae, Elatridae, Lampyridae, Melyridae
Acari	Phytoseiidae
Araneae	Linyphiidae, Lycosidae, Micryphantinae
Opiliones	Ischyropsalididae,Nemastomatidae Phalangiidae, Sclerosomatidae, Sabaconidae, Travuniidae, Trogulidae
Chilopoda	Cryptopidae, Geophilidae, Henicopidae, Himantariidae, Linotaeniidae, Lithobiidae, Pseudoannolenidae, Scolopendrellidae
Heteroptera	Anthocoridae, Miridae, Nabidae, Pentatomidae, Reduviidae
Dermaptera	Chelisochidae, Forficulidae, Labiduridae, Labiidae
Neuroptera	Chrysopidae, Hemerobiidae
Diptera	Asilidae, Cecidomyiidae, Chamaemyiidae, Dolichopodidae, Empididae, Syrphidae, Sciomyzidae
Hymenoptera	Numerous families, including Aphidiidae, Braconidae, Chalcididae, Dryinidae, Formicidae, Tenthredinidae, Vespulidae
Thysanoptera	Aeolothripidae, Thripidae

In general, pest impacts are much rarer in the Holarctic ecosystems. For example in Britain the most severe pests of ryegrass are the larvae of leather jackets, *Tipula* spp. L. (Diptera: Tipulidae) (Blackshaw 1984), yet they attract very little attention compared to the range of severe pasture pests in New Zealand (e.g., Barker et al. 2017). Overall, the lack of literature on pest impacts on Holarctic pasture indicates a lack of serious problems.

### Opportunity and Measures Taken

The widely diverse Holarctic predatory fauna (e.g., Table 1) may be considered to be characteristic of boundary and open fields (e.g., Collins et al. 2002). Luff (1966) sampled tussock grassland in the United Kingdom and recovered 198 species of Staphylinidae and Carabidae (Coleoptera) that were among the most abundant families. However, in spite of such natural enemy diversity in the vicinity, some crops (especially Gramineae) are repeatedly affected by pest impacts, particularly where there has been extensive clearance of remnant indigenous vegetation (e.g., Rusch et al. 2016). Recognition of this highlighted the opportunity for conservation biological control and its application and successes have been well-documented by Heimpel and Mills (2017). Much of this work has been focused on habitat analysis and variations of ‘ecological engineering’ (sensu Evans 2005).

Research into this subject was extensive in the 1990s, particularly in Britain. This was and remains, focused on how biological control impacts may best be conserved and fostered. The provision of floral resources for access to nectar, particularly for parasitoids, is well known (e.g., Pickett et al. 1998, Tylianakis et al. 2004, Heimpel and Mills 2017). More broadly a large amount of work was based on investigation into the deliberate provision of overwintering sites and refuges from disturbance (e.g., Thomas et al. 1992, Landis et al. 2000, Tscharntke et al. 2008, Rusch et al. 2010). Dennis and Fry (1992) showed how field margins can influence species richness and may enhance predators of crop pests in adjacent crops in the spring. Based on such observations, Thomas et al. (1991) described the development of raised banks of earth covered with rough tussocky grass to create ‘islands of complexity’ (later known as ‘beetle banks’) (e.g., MacLeod et al. 2004). These were often placed both at the margin and in the center of cereal fields to replace the favorable aspects provided by lost hedgerows and could support densities of predators similar to, or greater, than those found in conventional hedgerows (Thomas et al. 1991, 1992; Collins et al. 1996). Collins et al. (2002) provided quantitative evidence that beetle banks in the middle of a cereal field can have a significant effect on reducing aphid populations although this effect vanished beyond 83 m from the bank. In considering parasitoids specifically, Holland et al. (2012) discussed their contribution to integrated pest management. Furthermore, Thies et al. (2011) and Dainese et al. (2017) observed that the benefits of epigeal and aerial natural enemies were additive. As with the predators, Jonsson et al. (2008) noted the potential to enhance parasitoid impacts through targeted habitat management. Holarctic predatory spiders have also been identified as useful in conservation biological control potential. For example Nyffeler and Benz (1987) noted that in undisturbed grassland and forest ecosystems, spiders can play an important ecological role as predators of insects and other invertebrates. Of the spiders in Europe with known biological control potential, *Tenuiphantes tenuis* (Blackwall) (Araneae: Liniphyidae) is well-known. This species is amenable to enhancement through habitat manipulation (e.g., Alderweireldt 1994) and uses a ballooning mode of dispersal (e.g., Forster and Forster 1973) to re-colonize areas distant from undisturbed habitats (Samu et al. 1996).

Overall, van Emden and Williams (1974) and Dennis and Wratten (1991) concluded that the enhancement of polyphagous predators Holarctic in crops provided an economic justification for having field-margin habitats in farm landscapes. However, there remains uncertainty about aspects of conservation biological control because of the spiraling complexity found in such systems (Heimpel and Mills 2017). Moreover, when subjected to international meta-analysis, conservation biological control has not consistently been shown to confer beneficial effects on agricultural production (e.g., Karp et al. 2018) (discussed subsequently).

### New Zealand

Contrasting with the extensive effort in conservation biological control in the Holarctic, the potential for conservation biological control in New Zealand pasture is limited. This is despite the recognized importance of biodiversity and ecosystem services across New Zealand’s primary industries per se (e.g., Meurk and Swaffield 2000, Norton and Miller 2000, Blackwell et al. 2008, Lee et al. 2008, Moller et al. 2008).

There have, of course, been studies into the natural enemies and their refugia across New Zealand’s grassland ecosystems (e.g., Barratt and Patrick 1987, Dennis et al. 1998, Derraik et al. 2001, Barratt et al. 2005, Murray et al. 2006, Barratt et al. 2009; 2012, Tozer et al. 2014, Tozer et al. 2016). More specifically, Tozer et al. (2016) sampled invertebrates from five regions of New Zealand using a suction sampler and soil sampling and demonstrated that composition of natural enemy communities varied with pasture botanical diversity. However, it has remained uncertain whether New Zealand pasture has a useful range predators sufficient for conservation biological control. Thus for comparative purposes, Goldson et al. (2017) conducted a preliminary analysis of British natural enemy diversity lists in cropping rotations as developed by Ellis et al. (2014). For many taxa, the evidence has pointed to the number natural enemies in New Zealand being far lower than Britain (Goldson et al. 2017). For example, in Britain there are 274 species of Syrphidae (Diptera) (Ellis et al. 2014), compared to about 45 in New Zealand; estimates in pasture itself have been as low as 10 (Goldson et al. 2017), but even this is probably too high. In a comprehensive study in the Canterbury region of New Zealand, Curtis et al. (2019) found only syrphids; these being *Melangyna fasciatum* (Macquart) and *M. novazelandiae* (Macquart). Given that there are so few data on natural enemies in New Zealand pasture, it is also helpful to refer to comparative data from New Zealand’s arable cropping ecosystems. For example, Sivasubramaniam et al. (1997) examined the species composition, abundance and activity of predatory fauna in New Zealand carrot fields. It was found that the epigeal fauna was dominated by spiders, staphylinids (Coleoptera), and harvestmen (Arachnida) and relative to their incidence in European carrot crops (Boivin and Hance 1994), carabid beetles occurred in low numbers. Similarly, Wratten and Pearson (1982) found only very low populations of carabids in New Zealand in sugar beet crops, again, in marked contrast to Britain (e.g., Jepson 1982).

Simplistic comparison of natural enemy faunal lists from the Holarctic cropping systems (Ellis et al. 2014) versus what is found in New Zealand pasture has obvious limitations. However, as already noted, with regard to New Zealand pasture, the number of predator and parasitoid species listed is likely to be an over-estimate of potential as there is so little movement of native species out of the indigenous ecosystems. Thus, despite natural enemy species appearing in New Zealand’s indigenous faunal lists, they are often not found in New Zealand’s pasture.

In contrast to pasture’s paucity of exotic pest enemies, the New Zealand-indigenous pasture pests have a typical cohort of co-evolved indigenous natural enemies. For example, *C. giveni* is parasitized by the tachinid *Proscissio cana* Hutton (Diptera: Tachinidae) (Thomas 1963). The *Wiseana* spp. are attacked by several tachinids including *Occisor versutus* Hutton, *Ctenophorocera usitata* (Hutton), and *Plagiomyia sp.* Curran, as well as the ichneumonids (Hymenoptera: Ichneumonidae) *Pterocormus lotatorius* F. and *Degithina decepta* Smith (Eyles 1965). While at first sight such predators appear promising, this is not the case. The range of *Costelytra* spp. and *Wiseana* spp. parasitoids are restricted to pasture areas adjacent to indigenous shrublands that provide obligate nectar resources (Eyles 1965). With regard to pathogens *Costelytra* spp. and *Wiseana* spp. also have their own suites of soil-borne pathogens (Bourner et al. 1996). The native scarabs are susceptible to the buildup of in the soil-borne pathogenic bacteria and protozoans (Popay 1992, Jackson and O’Callaghan 2006, Hurst et al. 2014), whereas the *Wiseana* spp. are vulnerable to nucelopolyhedrosis, granulosis, entomopox and iridescent viruses (e.g., Moore et al. 1974). Also both pest groups can be infected by a range of fungal pathogens (Glare et al. 1993). While these microbial species are important in regulating populations of both pests, delayed density-dependent action prevents useful pest suppression before significant pasture damage has occurred.

### Opportunity and Measures Taken

In spite of the very limited assortment of natural enemies in New Zealand pastures, there may be limited opportunity for some conservation biological control; mainly using exotic species. However, basic information on their biology and ecology is lacking and what there is, shows little potential. Moreover, population dynamics analyses into New Zealand’s pasture pests have indicated strong density-dependent compensation (e.g., Barker et al. 1989a, Barlow and Goldson 1993, Barker and Addison 2006, Goldson et al. 2011, Barker 2013), which means that natural enemy attack rates have to be very high to achieve any pest suppression.

In life table studies, Barker et al. (1989a) noted predation of adult *L. bonariensis* by *Thyreocephalus orthodoxus* (Olliff) (Coleoptera: Staphylinidae) and the linyphiid spider *Mynoglenus subdola* (Cambridge) (Arachnidae: Linyphiidae), but they were unable to detect any suppressive effect. Similarly, they observed *L. bonariensis* egg predation by an exotic ant, *Ponera eduardi* Forel (Hymenoptera: Formacidae) but irrespective, the weevil has remained a potent pest.

There has also been some attention to the diversity of spiders as potential natural enemies of invasive species in pastures (e.g., Topping and Lövei 1997, Forster and Forster 1999, Mclachlan and Wratten 2003, Vink and Kean 2013). These again were found to occur in only half the numbers found in British pastures (Topping and Lövei 1997) and furthermore, Forster and Forster (1999) found that >95% of the population was endemic and as such, remained their native ecosystems. There is, therefore, little spider opportunity for conservation biological control in pasture. A possible exception is *T. tenuis* which because of its ballooning dispersal occurs across New Zealand’s agricultural habitats (Sivasubramaniam et al. 1997, Mclachlan and Wratten 2003, Vink and Kean 2013). Vink and Kean (2013) found that this spider attacks *L. bonariensis* and could be amenable to enhancement.

Also there may be some potential to enhance exotic parasitoid impacts through targeted habitat management (Tylianakis et al. 2004). Vattala (2005) and Vattala et al. (2006) showed the possibility of enhancing *M. hyperodae* efficacy through the provision of energy via nectar-bearing plants. However, suitable flowering species are absent in New Zealand pasture and clover flower morphology is such that its nectar is unavailable to *Microctonus* spp. (Vattalla et al. 2006). Furthermore, any deliberately introduced parasitoid food plants into the fields are very unlikely to survive under New Zealand pasture grazing pressure (e.g., Tozer et al. 2016, Gerard et al. 2018). Floral resources sown in the border regions of fields supported *M. hyperodae* but the numbers declined rapidly with increasing distance from the boundary reaching the lowest densities 7 m out (Vattalla et al. 2006).

While soil-borne pathogens have been shown to be important in regulation of native scarabids and hepialids (as discussed earlier), they are not particularly important in regulating exotic pest populations. Apparently, these pathogens have evolved specifically with indigenous species and are not generalist enough to affect the invasive species.

Against such background of limited potential for conservation biological control, consideration has also been given to the prospect of increasing the biodiversity of New Zealand pasture and surrounding habitat to attract natural enemies. In British grassland ecosystems, Curry (1994) showed that including herbs in a grass–legume mix can alter sward architecture sufficiently to provide an expanded range of invertebrate food sources. Similarly, in New Zealand, Norton and Miller (2000), Bowie et al. (2016), and Curtis et al. (2019) have suggested that areas surrounding New Zealand paddocks could provide habitats for natural enemies that could suppress grassland pest species. Bowie et al. (2014) conducted a survey of wire fence and hedge field margins in Canterbury and identified which taxa appeared to be sufficiently abundant to potentially impact pest populations. As a follow-up, Curtis et al. (2019) conducted a systematic study using plantings of native species in the vicinity of pasture but essentially, showed no (immediate) effect on the natural enemies nearby. Tozer et al. (2016) and Gerard et al. (2018) both tested whether biotic resistance to invasive pests could be increased by the inserting plant diversity into the pasture itself to attract useful natural enemies.

Tozer et al. (2016) surveyed the effects of combinations of ryegrass, clover, chicory, and plantain in pasture and concluded that any (ephemeral) changes in natural enemy numbers were unlikely to result in agronomic or other measurable effects. Tozer et al. (2016) also noted that such added plant species, with the possible exception of plantain, did not persist in intensively grazed New Zealand pasture. Gerard et al. (2018) in a 1-yr study, inserted combinations of timothy, chicory, tetraploid perennial ryegrass, and cocksfoot into small ryegrass-clover plots to test for any effect on of lacewings (Neuroptera) and parasitoids; the study revealed minor, if any, effects natural enemy abundance. Again chicory, cocksfoot, and especially timothy, failed to persist under grazing-pressure. Laliberté and Tylianakis (2012) researched the effect of intensive pastoral use mechanistically in a 20-yr experiment and found that grazing and fertilization drove plant communities towards a limited set of traits (e.g., high specific leaf area), which further reduced any sought-after diversity. Finally, Pembleton et al. (2015) pointed out that to include pasture-enriching species generally requires modification to the management of dairy pasture. This is likely to involve nitrogen fertilizer use and grazing to ensure that additional herbage species remain productive and persistent.

As a counter to poor prospects for conservation biological control in New Zealand pastures, it is often suggested that extensive importation of plants and insects could be used to increase the natural enemy biodiversity such that they would more closely resemble those of the Holarctic. However, this would be impractical as the biosecurity and biodiversity impacts would be massive (e.g., Barratt and Moeed 2005). Ironically, both Power (1968) and Lövei (1990) have made the point that New Zealand’s intensive pastures show virtually no evolutionary history which, in turn, offers great potential for studying the ecological aspects of community organization. This opportunity has subsequently been recognized and realized adding to biological control theory (e.g., Barlow and Goldson 1993, Gerard et al. 2010, Goldson et al. 2011).

## Interactions of *Epichloë* Endophyte Pasture Plant Resistance and Biocontrol

As alluded to above, *Epichloë* endophyte resistance to *L. bonariensis* and subsequently other pest species has had a major impact on New Zealand pasture pest management. This source of resistance was discovered in New Zealand’s ryegrass pastures (Mortimer et al. 1982; Prestidge et al. 1982; Barker et al. 1983, 1984) and the *Epichloë* endophyte ‘wild-type’ strain was found to be prevalent across New Zealand (Burgess and Easton 1986, Easton 1999). In hindsight, Hume and Barker (2005) noted that plant-breeding efforts had probably unwittingly selected endophytic plants. Since the initial discovery remarkable progress has been made in understanding its characteristics and *Epichloë* endophyte and developing specific and useful strains (e.g., Johnson et al. 2013, Kauppinen et al. 2016). However, although very important for New Zealand pastoral agriculture, *Epichloë* endophyte does not protect all *Lolium* spp. and *Lolium* hybrids against all pests (Popay et al. 2017). Ruppert et al. (2017) also found that protection of early-stage *Epichloë*-infected ryegrass seedlings is incomplete due to delayed alkaloid expression in emergent seedlings.

 Until the loss of *M. hyperodae* as a control agent, there was some evidence of negative interaction between the parasitoid and *Epichloë* endophyte as shown by Barker and Addison (1993), Goldson et al. (2000), and Bultman et al. (2003). However, in a large field experiment, Goldson et al. (2015a) found no evidence of such negative interaction. There is indication that such variation in effects may be influenced by selected-for *Epichloë* strains that differ in their metabolite composition.

## Theoretical Considerations Relating to Conservation Biological Control in the Holarctic and New Zealand Grassland Ecosystems

Numerous studies have highlighted the negative effects of habitat fragmentation on conservation biological control (e.g., Kruess and Tscharntke 1994). In particular, in his classical work, Root (1973) explored the effect of experimental differences in the area of host-plant resources on the biological control suppression of herbivores present. His ‘resource concentration’ hypothesis states that herbivores are more likely to find and remain on hosts that are growing in dense or nearly pure stands and that the most specialized of the herbivores may frequently attain the highest relative densities in these simple environments. This leads to plant biomass becoming concentrated in a few species with correspondingly reduced herbivore diversity. Root’s (1973) alternative (‘i.e., enemies’) hypothesis posited that low diversity of herbivores in pure stands could not support a diverse natural enemy guild and such a paucity of enemies can allow herbivores to attain high densities. This ‘enemies’ hypothesis generated interest in habitat management in order to enhance the range of natural enemies. This thinking eventually became central to the thinking about conservation biological control (e.g., Landis et al. 2000). Such growing interest in the biodiversity-ecosystem functioning relationship led to mechanistic insights into the synergistic effects of diverse natural enemy guilds (e.g., Ives et al. 2005, Snyder et al. 2006). This was pursued further by Tylianakis et al. (2008), who empirically used the results of various field studies to show that the extent of benefit of natural enemy diversity for prey suppression is relatively weak in homogenous environments, such as those typified by New Zealand’s high-intensity pastures. Further, Tylianakis and Romo (2010) noted that research emphasis had often been focused on those predator traits that maximize complementarity in prey suppression. Conversely, comparatively less attention has been paid to prey traits, or habitats that maximize the value of any predator diversity effects. These workers argued that those pests with patchy distributions, or complex life cycles, maybe more strongly suppressed when there is substantial predator diversity.

The high density of *L. bonariensis* populations in New Zealand’s pasture fulfills either or both of Root’s (1973) hypotheses that herbivores are more likely to find and remain on plant hosts that are growing in relatively pure stands. These relatively pure stands (of pasture) typically support little predator diversity leading to high densities of pest herbivores (i.e., often pests). The presence of *M. hyperodae* represents typical low predator diversity in low diversity pasture. Yet contrary to the Root (1973) hypotheses, this single species of parasitoid had a major effect on reducing the herbivore (weevil) population. This departure from what has been found elsewhere may well be partly explained by the single, deliberate introduction of exotic *M. hyperodae* into a nonevolved ecosystem. Indeed, since the onset of *L. bonariensis* resistance to *M. hyperodae* and the recovery of higher weevil populations (Tomasetto et al. 2017), this dynamic ironically now fulfills Root’s (1973) enemies hypothesis.

A review by Tscharntke et al. (2012) showed in habitats comprising very low complexity, natural enemy enhancement through habitat manipulation is ineffective as there are few enemies to be enhanced. Conversely, they also noted that measures to enhance biological control in species-rich ecosystems makes little or no difference because of the incumbent diversity. Thus, based on these two extremes, conservation biological control interventions are only likely to work in ecosystems with ‘intermediate landscape complexity.’ The unique nature of New Zealand’s pasture ecosystem clearly puts it at the very low complexity extensive end of the spectrum.

A meta-analysis of global data showed that overall biological control effects on production were heterogeneous, i.e., the effects on pest suppression were inconclusive (Karp et al. 2018). However, models from the Holarctic realms generally explained more variation in pest abundance and activity than those from other areas. This difference may be attributable to variation in latitude and data quality, particularly from the tropical regions. Also it is notable that intensive farming systems in the non-Holarctic regions are often founded on relatively few crop species developed in and imported from the Holarctic. As in New Zealand, these crops are cultivated in alien ecosystems that may be damaged by accidentally introduced exotic pest species, some of which benefit from enemy release. Also, because of evolutionary isolation, there is often an absence of rich functional native natural enemy diversity in these regions and what there is, tends to stay in its indigenous ecosystems. Thus, the ‘imported agricultural systems’ are more susceptible to pest impacts than those in the Holarctic, where there already exists a background complex of active natural enemies that are amenable to manipulation using conservation biological control.

The Island Resource Allocation hypothesis developed by Kay and Wratten (2003) may also explain why New Zealand’s native natural enemies have had little or no impact on exotic pasture pests. Generally, it is thought that mature island indigenous tend to be susceptible to invasion by continental species primarily because of the relative availability of unfilled niches and the nature of evolution-in-isolation of insular biota (Primack 1993). However, Brockerhoff et al. (2010) contended that this interpretation does not necessarily fit well with the observed New Zealand situation. They posited that plants belonging to a large geographical range can rely on top-down suppression of damaging defoliators via a well-developed and mobile natural enemy fauna of parasitoiods and predators. Conversely, when such a natural enemy resource is constrained, such as in restricted land areas, then plants tend to develop bottom-up defences against herbivores via resistance. The implication here is that New Zealand has a relative paucity of ‘top down’ natural enemies in its indigenous habitats. Finally, there is a lack of a ‘rescue effect’ for New Zealand as an island ecology, such that when natural enemies are lost or displaced through major perturbations (e.g., glaciation effects), there is little prospect of replenishment from surrounding territories.

## New Zealand Pastures: Not What They Seem

New Zealand’s agricultural landscapes may visually resemble those of the Holarctic. Yet, as discussed, its pastoral ecology is extraordinary and unique; it abounds with anomalous and incongruous ecological characteristics that could at least partly explain poor pasture persistence in New Zealand (e.g., Parsons et al. 2011).

Also, unlike the Holarctic, New Zealand field studies do not indicate that enhancing species diversity within or around New Zealand pastures will increase biotic resistance to invasive pests, let alone their suppression. At the same time, the contribution described in this paper, highlights some other interesting ecological trade-offs that can be discerned at a number of levels. For example, it has become apparent that biological control in New Zealand’s grasslands can be double-edged (e.g., Goldson et al. 2014a). The lack of New Zealand pasture ecosystem complexity and concomitant sparseness of effective natural enemies, either in the pasture or in nearby indigenous habitats, all point to high susceptibility to invasion and scant potential for conservation biological control. Conversely, it is this same lack of complexity that also explains the singular success of the *Microctonus* spp. biological control programs discussed earlier; the introduced parasitoids have thrived through their own enemy release. Finally, it has become apparent that such spectacular biological control can become unstable (e.g., Tomasetto et al. 2017) through exceptional and highly focused selection pressure resulting from minimal interspecific competition and predation.

These observations raise questions about how to counter or exploit such unusual circumstances. Certainly, there is need to understand more of the ecology and genetics behind the importation of a successful biological control agents to anticipate possible resistance occurring in similar biological control systems. This particularly applies to *S. obsoletus* (also currently suppressed by a single parthenogenetic *Microctonus* spp. (Goldson et al. 2005b). In the case of natural enemies there are opportunities for increasing genetic diversity through further field searches and collections of sexually reproducing populations. Additionally, it may be possible to alter the reproductive physiology of a parthenogenetic species to allow expression of male-coding genes, thereby allowing ongoing selection of more virulent strains through sexual recombination. While Goldson et al. (2003) found that crossing the sexually reproducing *M. aethiopoides* populations from Europe and Morocco resulted in reduced biological control potential in the offspring, the potential for further crossing and back-crossing remains.

The claim here that New Zealand grassland pastures are unique in their lack of complexity may be open to challenge. Large areas of high production grassland exist in countries such as Brazil and Chile, where pest outbreaks can occur despite the presence of co-evolved indigenous natural enemies. However, reports of continuous pest impacts are uncommon in these ecosystems.

This contribution in no way seeks to deny the ecological and aesthetic importance of maintaining wherever possible, and even increasing, the biodiversity of New Zealand’s farmed landscapes. Landscape diversity greatly improves ecosystem services, including pollination. Other services include the interception of soil-borne contaminants through nitrate leaching, phosphate loss and sediment movement, as well as increasing terrestrial and aquatic biodiversity (e.g., Daigneault et al. 2017). At the same time New Zealand’s indigenous biodiversity is protected, including its avifauna as well as the creation of more attractive landscapes (Norton and Miller 2000). However, in spite of such major benefits, this paper points to the probability that where production is a primary goal, increasing botanical diversity within New Zealand’s pasture and its surrounding areas, will probably not improve biotic resistance to invasive species or lead to pest suppression. As discussed, New Zealand’s native natural enemy fauna largely remains in its indigenous ecosystems.

### Conclusion

After a century of agronomic and pest management research, it is apparent that New Zealand’s intensively managed imported pasture ecosystems are unique in their lack of evolved complexity and its effects. This contribution outlines the occurrence and interaction of novel associations of phylogenetically remote species that arguably lead to unusual or unexpected ecological phenomena such as the appearance of pest resistance to a biological control agent.

Through the lack of biotic resistance, the extreme vulnerability of New Zealand intensive pastoral farmlands to invasive species has long been apparent. Typical of this has been the impacts arising from three invasive weevil species, that are of minimal concern in their centers-of-origin, but have caused massive damage to New Zealand pastures (and lucerne forage) by building up to densities far beyond those found in their native ranges. However, the introduction of three braconid biological control agents was highly and unexpectedly effective against these weevils with the odd of this occurring being 1:1,000. It is plausible that these braconids, as with the target pests, encountered little or no biotic resistance, with enemy release allowing parasitism to exceed 90%. These selection pressures have been such that resistance has developed in the weevil pest, *L. bonariensis* to the parasitoid *M. hyperodae*. Such occurrences are in stark contrast to conservation biological control, which apparently shows scant opportunity for encouraging the movement of natural enemies from their native ecosystems into New Zealand’s species-alien pastures. As a consequence of such considerations, the resource of introduced exotic natural enemies must be conserved by managing their genetic diversity by various means including selecting for efficacy. This calls for a better understanding of the genetics of the pest-parasitoid diversity and the expansion of the natural enemies’ genetic diversity. Exotic pest management in the New Zealand pasture ecosystems is neither intuitive nor obvious; the dynamic is unlike that elsewhere. Any lessons learned have to be New Zealand-based. Such understanding would serve to advance biological control theory in more complex ecosystems elsewhere.
